# Crystal structure of morpholin-4-ium cinnamate

**DOI:** 10.1107/S2056989015019179

**Published:** 2015-10-17

**Authors:** Graham Smith

**Affiliations:** a Science and Engineering Faculty, Queensland University of Technology, GPO Box 2434, Brisbane, Queensland 4001, Australia

**Keywords:** crystal structure, salt, morpholinium, cinnamate, hydrogen bonding

## Abstract

In the anhydrous salt formed from the reaction of morpholine with cinnamic acid, C_4_H_10_NO^+^·C_9_H_7_O_2_^−^, the acid side chain in the *trans*-cinnamate anion is significantly rotated out of the benzene plane [C—C—C— C torsion angle = 158.54 (17)°]. In the crystal, one of the the aminium H atoms is involved in an asymmetric three-centre cation–anion N—H⋯(O,O′) *R*_1_^2^(4) hydrogen-bonding inter­action with the two carboxyl­ate O-atom acceptors of the anion. The second aminium-H atom forms an inter-species N—H⋯O_carboxyl­ate_ hydrogen bond. The result of the hydrogen bonding is the formation of a chain structure extending along [100]. Chains are linked by C—H⋯O inter­actions, forming a supra­molecular layer parallel to (01-1).

## Related literature   

For background on morpholine compounds and the structure of an aliphatic morpholine salt, see: Kelley *et al.* (2013 ▸). For the structures of analogous morpholinate salts of some aromatic acid analogues, see: Chumakov *et al.* (2006 ▸); Ishida *et al.* (2001*a* ▸,*b* ▸,*c* ▸); Smith & Lynch (2015 ▸).
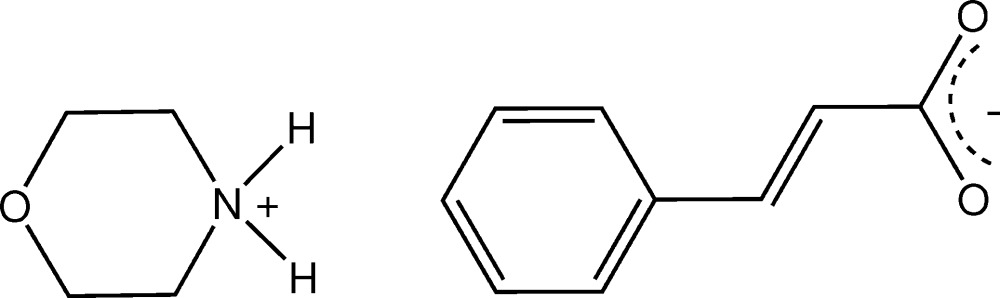


## Experimental   

### Crystal data  

C_4_H_10_NO^+^·C_9_H_7_O_2_^−^
*M* *_r_* = 235.27
Triclinic, 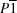

*a* = 5.7365 (7) Å
*b* = 9.7526 (10) Å
*c* = 11.7760 (11) Å
α = 103.270 (8)°
β = 93.468 (9)°
γ = 105.493 (10)°
*V* = 612.69 (12) Å^3^
*Z* = 2
Mo *K*α radiation
μ = 0.09 mm^−1^
*T* = 200 K
0.52 × 0.24 × 0.05 mm

### Data collection  

Oxford Diffraction Gemini-S CCD-detector diffractometer
Absorption correction: multi-scan (*CrysAlis PRO*; Agilent, 2014 ▸) *T*_min_ = 0.965, *T*_max_ = 0.990
4253 measured reflections
2393 independent reflections
1860 reflections with *I* > 2σ(*I*)
*R*_int_ = 0.023

### Refinement  

*R*[*F* ^2^ > 2σ(*F* ^2^)] = 0.043
*wR*(*F* ^2^) = 0.100
*S* = 1.01
2393 reflections
160 parameters
2 restraints
H atoms treated by a mixture of independent and constrained refinement
Δρ_max_ = 0.15 e Å^−3^
Δρ_min_ = −0.15 e Å^−3^

### 

Data collection: *CrysAlis PRO* (Agilent, 2014 ▸); cell refinement: *CrysAlis PRO*; data reduction: *CrysAlis PRO*; program(s) used to solve structure: *SIR92* (Altomare *et al.*, 1993 ▸); program(s) used to refine structure: *SHELXL97* (Sheldrick, 2008 ▸) within *WinGX* (Farrugia, 2012 ▸); molecular graphics: *PLATON* (Spek, 2009 ▸); software used to prepare material for publication: *PLATON*.

## Supplementary Material

Crystal structure: contains datablock(s) global, I. DOI: 10.1107/S2056989015019179/tk5397sup1.cif

Structure factors: contains datablock(s) I. DOI: 10.1107/S2056989015019179/tk5397Isup2.hkl

Supporting information file. DOI: 10.1107/S2056989015019179/tk5397Isup3.cml

**Fig. 1.**
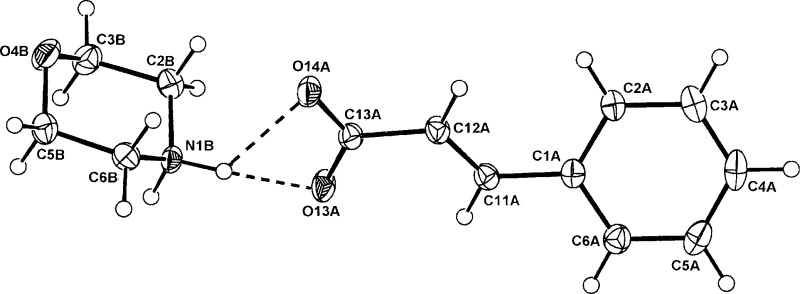
The atom-numbering scheme and the molecular conformation of the morpholinium anion (B) and the cinnamate cation (A) in the title salt, with displacement ellipsoids drawn at the 40% probability level. The cation–anion hydrogen bonds are shown as dashed lines.

**Fig. 2.**
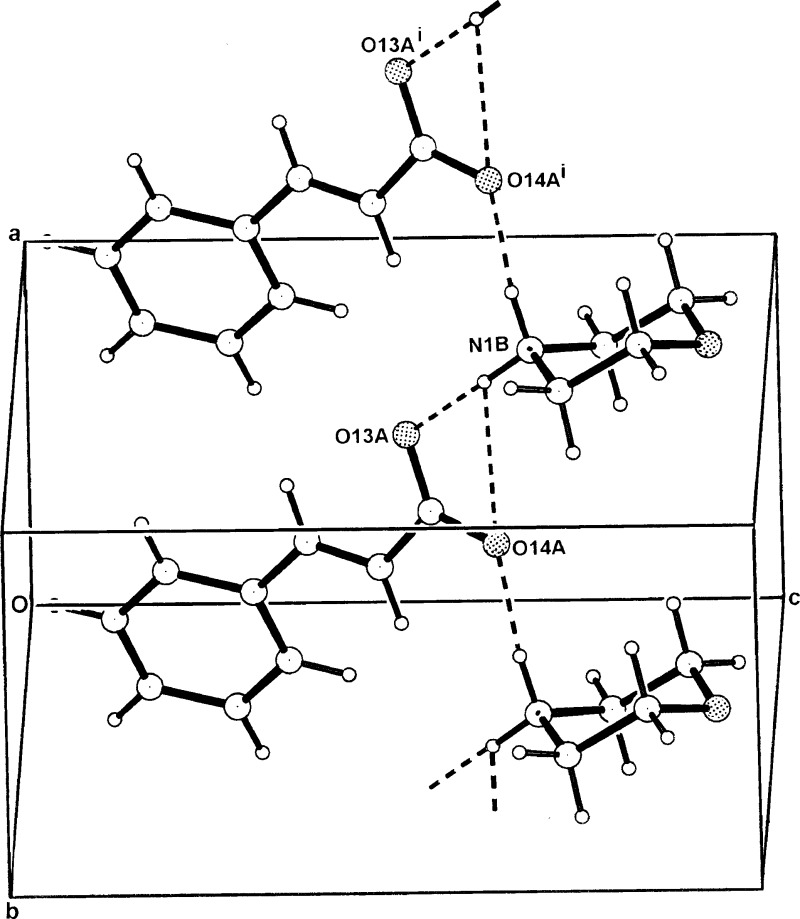
The one-dimensional hydrogen-bonded polymeric structure extending along a. For symmetry codes, see Table 1.

B A . DOI: 10.1107/S2056989015019179/tk5397fig1.tif
The atom-numbering scheme and the mol­ecular conformation of the morpholinium anion (*B*) and the cinnamate cation (*A*) in the title salt, with displacement ellipsoids drawn at the 40% probability level. The cation–anion hydrogen bonds are shown as dashed lines.

a . DOI: 10.1107/S2056989015019179/tk5397fig2.tif
The one-dimensional hydrogen-bonded polymeric structure extending along *a*. For symmetry codes, see Table 1.

CCDC reference: 1430629

Additional supporting information:  crystallographic information; 3D view; checkCIF report

## Figures and Tables

**Table 1:** Hydrogen-bond geometry (, )

*D*H*A*	*D*H	H*A*	*D* *A*	*D*H*A*
N1*B*H11*B*O14*A* ^i^	0.94(2)	1.77(2)	2.7052(17)	170(2)
N1*B*H12*B*O13*A*	0.94(2)	1.73(2)	2.6643(17)	172(2)
N1*B*H12*B*O14*A*	0.94(2)	2.57(2)	3.1868(17)	123(1)
C4*A*H4*A*O4*B* ^ii^	0.95	2.46	3.393(2)	167
C6*B*H62*B*O13*A* ^iii^	0.99	2.37	3.234(2)	145

Symmetry codes: (i) 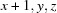; (ii) 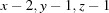; (iii) 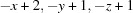.

## supplementary crystallographic information

### S1. Comment

Morpholine (tetrahydro-2*H*-1,4-oxazine) forms salts with organic acids, and the crystal structures of a limited number of these with either aliphatic acids, *e.g.* the acetate (Kelley *et al.*, 2013) or aromatic acids, *e.g.* the 4-nitrobenzoate (Chumakov *et al.*, 2006), have been reported. With the salts of the aromatic acids, particularly those with non-associative substituent groups, cation–anion *N*—*H*···O_carboxyl_ hydrogen-bonding interactions generate either one-dimensional chains or discrete cyclic heterotetrameric structures. In the present work, the title morpholinium salt of cinnamic acid, C_4_H_10_NO^+^ C_9_H_7_O_2_^-^ was prepared and its structure is reported herein.

The asymmetric unit of the title salt comprises a morpholinium cation (*B* and a cinnamate anion (*A*), (Fig. 1). In the *trans*- cinnamate anion, the acid side chain is significantly rotated out of the benzene plane [defining torsion angle C6*A*—C1*A*—C11*A*— C12*A* = 158.54 (17)°]. In the crystal, a primary asymmetric three-centre *R*^2^_1_(4) N1*B*—*H*···(*O*,*O*')_carboxyl_ hydrogen-bonding interaction is present [N···O = 2.6643 (17) and 3.1868 (17) Å] (Table 1). The hydrogen-bonding extension involves the second aminium H atom of the cation to the carboxyl O14*A*^i^ acceptor of the anion, resulting in a one-dimensional ribbon structure extending along *a* (Fig. 2). Present also in the structure are minor weak inter-unit C—H···O interactions. C4*A*—H···O4*B*^ii^; C6*B*—H··· O13*A*^iii^. No π–π interactions are present in the structure.

These ribbon structures are similar to those found in the morpholinium salt of one of the five isomeric chloro-nitrobenzoic acids (2,4-) (Ishida *et al.*, 2001*a*). In the other four isomers [(2,5-), (4,3-), (4,2-), (5,2-)] (Ishida, 2001*a*, 2001*b*, 2001*c*), the cyclic heterotetrameric structures are found. However, among a set of four morpholinium salts of phenoxyacetic acid analogues (Smith & Lynch, 2015), there are four one-dimensional polymers and one cyclic heterotetramer.

### S2. Experimental

The title compound was prepared by the dropwise addition of morpholine at room temperature to a solution of cinnamic acid (150 mg) in ethanol (10 ml). Room temperature evaporation of the solution gave an oil which was redissolved in ethanol, finally giving thin colourless plates of the title compound from which a specimen was cleaved for the X-ray analysis.

### S3. Refinement

Hydrogen atoms were placed in calculated positions [C—H_aromatic_ = 0.95 Å or *C*—H_methylene_ = 0.99 Å] and were allowed to ride in the refinements, with *U*_iso_(H) = 1.2*U*_eq_(C). The aminium H atoms were located in a difference-Fourier analysis and were allowed to refine with distance restraints [*d*(N—H) = 0.92 (2) Å and *U*_iso_(H) = 1.2*U*_eq_(N)

### Crystal data

**Table:**

C_4_H_10_NO^+^·C_9_H_7_O_2_^−^	*Z* = 2
*M**_r_* = 235.27	*F*(000) = 252
Triclinic, *P*1	*D*_x_ = 1.281 Mg m^−^^3^
Hall symbol: -P 1	Mo *K*α radiation, λ = 0.71073 Å
*a* = 5.7365 (7) Å	Cell parameters from 1133 reflections
*b* = 9.7526 (10) Å	θ = 3.6–28.4°
*c* = 11.7760 (11) Å	µ = 0.09 mm^−^^1^
α = 103.270 (8)°	*T* = 200 K
β = 93.468 (9)°	Plate, colourless
γ = 105.493 (10)°	0.52 × 0.24 × 0.05 mm
*V* = 612.69 (12) Å^3^	

### Data collection

**Table:**

Oxford Diffraction Gemini-S CCD-detector diffractometer	2393 independent reflections
Radiation source: Enhance (Mo) X-ray source	1860 reflections with *I* > 2σ(*I*)
Graphite monochromator	*R*_int_ = 0.023
Detector resolution: 16.077 pixels mm^-1^	θ_max_ = 26.0°, θ_min_ = 3.2°
ω scans	*h* = −6→7
Absorption correction: multi-scan (*CrysAlis PRO*; Agilent, 2014)	*k* = −12→12
*T*_min_ = 0.965, *T*_max_ = 0.990	*l* = −14→14
4253 measured reflections	

### Refinement

**Table:**

Refinement on *F*^2^	Primary atom site location: structure-invariant direct methods
Least-squares matrix: full	Secondary atom site location: difference Fourier map
*R*[*F*^2^ > 2σ(*F*^2^)] = 0.043	Hydrogen site location: inferred from neighbouring sites
*wR*(*F*^2^) = 0.100	H atoms treated by a mixture of independent and constrained refinement
*S* = 1.01	*w* = 1/[σ^2^(*F*_o_^2^) + (0.0429*P*)^2^ + 0.0676*P*] where *P* = (*F*_o_^2^ + 2*F*_c_^2^)/3
2393 reflections	(Δ/σ)_max_ < 0.001
160 parameters	Δρ_max_ = 0.15 e Å^−^^3^
2 restraints	Δρ_min_ = −0.15 e Å^−^^3^

### Special details

**Table:**

Geometry. Bond distances, angles etc. have been calculated using the rounded fractional coordinates. All su's are estimated from the variances of the (full) variance-covariance matrix. The cell esds are taken into account in the estimation of distances, angles and torsion angles
Refinement. Refinement of F^2^ against ALL reflections. The weighted R-factor wR and goodness of fit S are based on F^2^, conventional R-factors R are based on F, with F set to zero for negative F^2^. The threshold expression of F^2^ > 2sigma(F^2^) is used only for calculating R-factors(gt) etc. and is not relevant to the choice of reflections for refinement. R-factors based on F^2^ are statistically about twice as large as those based on F, and R- factors based on ALL data will be even larger.

### Fractional atomic coordinates and isotropic or equivalent isotropic displacement parameters (Å^2^)

**Table:**

	*x*	*y*	*z*	*U*_iso_*/*U*_eq_	
O13A	0.72059 (18)	0.32720 (13)	0.51574 (9)	0.0370 (4)	
O14A	0.50422 (18)	0.43517 (12)	0.63746 (10)	0.0323 (4)	
C1A	0.0669 (3)	0.04440 (16)	0.29775 (13)	0.0256 (5)	
C2A	−0.1554 (3)	0.00183 (17)	0.34093 (15)	0.0299 (5)	
C3A	−0.3583 (3)	−0.09424 (18)	0.26731 (16)	0.0365 (6)	
C4A	−0.3450 (3)	−0.14842 (18)	0.14947 (16)	0.0388 (6)	
C5A	−0.1258 (3)	−0.10770 (18)	0.10580 (15)	0.0384 (6)	
C6A	0.0789 (3)	−0.01381 (17)	0.17959 (14)	0.0321 (5)	
C11A	0.2852 (3)	0.14762 (17)	0.37395 (14)	0.0262 (5)	
C12A	0.2907 (3)	0.24300 (17)	0.47379 (14)	0.0276 (5)	
C13A	0.5213 (3)	0.34254 (17)	0.54714 (13)	0.0258 (5)	
O4B	1.2058 (2)	0.63511 (13)	0.93100 (10)	0.0398 (4)	
N1B	1.0764 (2)	0.48489 (14)	0.68969 (11)	0.0253 (4)	
C2B	1.0246 (3)	0.40701 (18)	0.78354 (14)	0.0310 (5)	
C3B	1.2089 (3)	0.48633 (18)	0.89057 (14)	0.0354 (6)	
C5B	1.2676 (3)	0.71057 (18)	0.84191 (15)	0.0355 (6)	
C6B	1.0875 (3)	0.64183 (17)	0.73241 (14)	0.0298 (5)	
H2A	−0.16720	0.03930	0.42160	0.0360*	
H3A	−0.50810	−0.12330	0.29790	0.0440*	
H4A	−0.48570	−0.21320	0.09880	0.0470*	
H5A	−0.11570	−0.14440	0.02480	0.0460*	
H6A	0.22990	0.01130	0.14920	0.0390*	
H11A	0.43820	0.14510	0.34830	0.0310*	
H12A	0.14000	0.24890	0.50070	0.0330*	
H11B	1.227 (3)	0.4752 (17)	0.6663 (13)	0.0300*	
H12B	0.951 (3)	0.4376 (17)	0.6261 (12)	0.0300*	
H21B	1.03230	0.30470	0.75550	0.0370*	
H22B	0.85830	0.40330	0.80370	0.0370*	
H31B	1.17210	0.43540	0.95390	0.0420*	
H32B	1.37370	0.48430	0.87120	0.0420*	
H51B	1.43250	0.70830	0.82290	0.0430*	
H52B	1.27130	0.81480	0.87160	0.0430*	
H61B	0.92430	0.65020	0.74950	0.0360*	
H62B	1.13770	0.69400	0.67100	0.0360*	

### Atomic displacement parameters (Å^2^)

**Table:**

	*U*^11^	*U*^22^	*U*^33^	*U*^12^	*U*^13^	*U*^23^
O13A	0.0181 (6)	0.0508 (8)	0.0320 (7)	0.0093 (5)	−0.0013 (5)	−0.0075 (6)
O14A	0.0217 (6)	0.0368 (7)	0.0304 (6)	0.0073 (5)	−0.0001 (5)	−0.0048 (5)
C1A	0.0255 (8)	0.0211 (8)	0.0289 (9)	0.0061 (7)	−0.0022 (7)	0.0063 (7)
C2A	0.0265 (8)	0.0251 (9)	0.0334 (9)	0.0043 (7)	−0.0008 (7)	0.0035 (7)
C3A	0.0259 (9)	0.0273 (9)	0.0516 (12)	0.0036 (7)	−0.0029 (8)	0.0075 (8)
C4A	0.0375 (10)	0.0242 (9)	0.0453 (11)	0.0036 (8)	−0.0174 (8)	0.0026 (8)
C5A	0.0522 (11)	0.0299 (10)	0.0279 (9)	0.0099 (9)	−0.0062 (8)	0.0026 (8)
C6A	0.0349 (9)	0.0279 (9)	0.0312 (9)	0.0069 (8)	0.0013 (7)	0.0065 (7)
C11A	0.0213 (8)	0.0277 (9)	0.0294 (9)	0.0065 (7)	0.0021 (6)	0.0080 (7)
C12A	0.0191 (8)	0.0311 (9)	0.0303 (9)	0.0072 (7)	0.0019 (6)	0.0039 (7)
C13A	0.0213 (8)	0.0295 (9)	0.0261 (9)	0.0076 (7)	0.0007 (6)	0.0064 (7)
O4B	0.0518 (8)	0.0356 (7)	0.0241 (6)	0.0068 (6)	−0.0002 (5)	0.0001 (5)
N1B	0.0185 (6)	0.0305 (8)	0.0222 (7)	0.0066 (6)	−0.0013 (5)	−0.0007 (6)
C2B	0.0287 (8)	0.0269 (9)	0.0361 (10)	0.0057 (7)	0.0024 (7)	0.0085 (8)
C3B	0.0402 (10)	0.0363 (10)	0.0286 (9)	0.0101 (8)	−0.0011 (7)	0.0088 (8)
C5B	0.0380 (10)	0.0257 (9)	0.0351 (10)	0.0009 (8)	0.0011 (8)	0.0032 (8)
C6B	0.0296 (9)	0.0280 (9)	0.0321 (9)	0.0082 (7)	0.0045 (7)	0.0085 (7)

### Geometric parameters (Å, º)

**Table:**

O13A—C13A	1.258 (2)	C2A—H2A	0.9500
O14A—C13A	1.2553 (19)	C3A—H3A	0.9500
O4B—C3B	1.425 (2)	C4A—H4A	0.9500
O4B—C5B	1.424 (2)	C5A—H5A	0.9500
N1B—C2B	1.480 (2)	C6A—H6A	0.9500
N1B—C6B	1.480 (2)	C11A—H11A	0.9500
N1B—H11B	0.944 (18)	C12A—H12A	0.9500
N1B—H12B	0.943 (15)	C2B—C3B	1.503 (2)
C1A—C6A	1.390 (2)	C5B—C6B	1.501 (2)
C1A—C2A	1.396 (2)	C2B—H21B	0.9900
C1A—C11A	1.471 (2)	C2B—H22B	0.9900
C2A—C3A	1.381 (2)	C3B—H31B	0.9900
C3A—C4A	1.382 (3)	C3B—H32B	0.9900
C4A—C5A	1.382 (3)	C5B—H51B	0.9900
C5A—C6A	1.382 (2)	C5B—H52B	0.9900
C11A—C12A	1.314 (2)	C6B—H61B	0.9900
C12A—C13A	1.493 (2)	C6B—H62B	0.9900
			
C3B—O4B—C5B	109.75 (12)	C1A—C6A—H6A	120.00
C2B—N1B—C6B	111.05 (12)	C12A—C11A—H11A	117.00
C6B—N1B—H11B	110.9 (10)	C1A—C11A—H11A	117.00
C2B—N1B—H12B	107.7 (10)	C11A—C12A—H12A	118.00
H11B—N1B—H12B	109.8 (14)	C13A—C12A—H12A	118.00
C2B—N1B—H11B	107.0 (10)	N1B—C2B—C3B	109.50 (14)
C6B—N1B—H12B	110.3 (10)	O4B—C3B—C2B	110.91 (14)
C2A—C1A—C11A	121.67 (14)	O4B—C5B—C6B	111.36 (14)
C6A—C1A—C11A	120.00 (15)	N1B—C6B—C5B	109.46 (14)
C2A—C1A—C6A	118.33 (15)	N1B—C2B—H21B	110.00
C1A—C2A—C3A	120.55 (16)	N1B—C2B—H22B	110.00
C2A—C3A—C4A	120.46 (17)	C3B—C2B—H21B	110.00
C3A—C4A—C5A	119.55 (16)	C3B—C2B—H22B	110.00
C4A—C5A—C6A	120.21 (16)	H21B—C2B—H22B	108.00
C1A—C6A—C5A	120.88 (16)	O4B—C3B—H31B	109.00
C1A—C11A—C12A	126.79 (16)	O4B—C3B—H32B	109.00
C11A—C12A—C13A	123.45 (16)	C2B—C3B—H31B	109.00
O13A—C13A—O14A	123.98 (15)	C2B—C3B—H32B	109.00
O13A—C13A—C12A	118.14 (14)	H31B—C3B—H32B	108.00
O14A—C13A—C12A	117.87 (15)	O4B—C5B—H51B	109.00
C1A—C2A—H2A	120.00	O4B—C5B—H52B	109.00
C3A—C2A—H2A	120.00	C6B—C5B—H51B	109.00
C4A—C3A—H3A	120.00	C6B—C5B—H52B	109.00
C2A—C3A—H3A	120.00	H51B—C5B—H52B	108.00
C3A—C4A—H4A	120.00	N1B—C6B—H61B	110.00
C5A—C4A—H4A	120.00	N1B—C6B—H62B	110.00
C6A—C5A—H5A	120.00	C5B—C6B—H61B	110.00
C4A—C5A—H5A	120.00	C5B—C6B—H62B	110.00
C5A—C6A—H6A	120.00	H61B—C6B—H62B	108.00
			
C3B—O4B—C5B—C6B	61.19 (17)	C1A—C2A—C3A—C4A	−0.7 (3)
C5B—O4B—C3B—C2B	−61.29 (17)	C2A—C3A—C4A—C5A	1.0 (3)
C2B—N1B—C6B—C5B	54.09 (17)	C3A—C4A—C5A—C6A	0.2 (3)
C6B—N1B—C2B—C3B	−54.46 (17)	C4A—C5A—C6A—C1A	−1.8 (3)
C2A—C1A—C6A—C5A	2.1 (2)	C1A—C11A—C12A—C13A	178.94 (15)
C6A—C1A—C11A—C12A	158.54 (17)	C11A—C12A—C13A—O13A	−5.0 (2)
C11A—C1A—C6A—C5A	−178.16 (16)	C11A—C12A—C13A—O14A	175.97 (16)
C2A—C1A—C11A—C12A	−21.7 (3)	N1B—C2B—C3B—O4B	57.95 (17)
C6A—C1A—C2A—C3A	−0.8 (2)	O4B—C5B—C6B—N1B	−57.43 (18)
C11A—C1A—C2A—C3A	179.41 (16)		

### Hydrogen-bond geometry (Å, º)

**Table:**

*D*—H···*A*	*D*—H	H···*A*	*D*···*A*	*D*—H···*A*
N1*B*—H11*B*···O14*A*^i^	0.94 (2)	1.77 (2)	2.7052 (17)	170 (2)
N1*B*—H12*B*···O13*A*	0.94 (2)	1.73 (2)	2.6643 (17)	172 (2)
N1*B*—H12*B*···O14*A*	0.94 (2)	2.57 (2)	3.1868 (17)	123 (1)
C4*A*—H4*A*···O4*B*^ii^	0.95	2.46	3.393 (2)	167
C11*A*—H11*A*···O13*A*	0.95	2.48	2.812 (2)	101
C6*B*—H62*B*···O13*A*^iii^	0.99	2.37	3.234 (2)	145

Symmetry codes: (i) *x*+1, *y*, *z*; (ii) *x*−2, *y*−1, *z*−1; (iii) −*x*+2, −*y*+1, −*z*+1.
